# Kisspeptin-Activated Autophagy Independently Suppresses Non-Glucose-Stimulated Insulin Secretion from Pancreatic β-Cells

**DOI:** 10.1038/s41598-019-53826-7

**Published:** 2019-11-25

**Authors:** Chien Huang, Hao-Yi Wang, Mu-En Wang, Meng-Chieh Hsu, Yi-Hsieng Samuel Wu, Yi-Fan Jiang, Leang-Shin Wu, De-Shien Jong, Chih-Hsien Chiu

**Affiliations:** 1 Laboratory of Animal Physiology, Department of Animal Science and Technology, National Taiwan University, Taipei, 10617 Taiwan; 2 Department of Pathology, Duke University School of Medicine, Duke Cancer Institute, Duke University, Durham, NC 27514 USA; 3 Graduate Institute of Molecular and Comparative Pathobiology, School of Medicine, National Taiwan University, Taipei, 10617 Taiwan

**Keywords:** Biochemistry, Cell biology, Molecular biology, Endocrinology

## Abstract

Previous studies have demonstrated the important role of kisspeptin in impaired glucose-stimulated insulin secretion (GSIS). In addition, it was reported that the activation of autophagy in pancreatic β-cells decreases insulin secretion by selectively degrading insulin granules. However, it is currently unknown whether kisspeptin suppresses GSIS in β-cells by activating autophagy. To investigate the involvement of autophagy in kisspeptin–regulated insulin secretion, we overexpressed *Kiss1* in NIT-1 cells to mimic the long-term exposure of pancreatic β-cells to kisspeptin during type 2 diabetes (T2D). Interestingly, our data showed that although kisspeptin potently decreases the intracellular proinsulin and insulin ((pro)insulin) content and insulin secretion of NIT-1 cells, autophagy inhibition using bafilomycin A1 and Atg5 siRNAs only rescues basal insulin secretion, not kisspeptin-impaired GSIS. We also generated a novel *in vivo* model to investigate the long-term exposure of kisspeptin by osmotic pump. The *in vivo* data demonstrated that kisspeptin lowers GSIS and (pro)insulin levels and also activated pancreatic autophagy in mice. Collectively, our data demonstrated that kisspeptin suppresses both GSIS and non-glucose-stimulated insulin secretion of pancreatic β-cells, but only non-glucose-stimulated insulin secretion depends on activated autophagic degradation of (pro)insulin. Our study provides novel insights for the development of impaired insulin secretion during T2D progression.

## Introduction

Type 2 diabetes (T2D) is a metabolic disease that is highly correlated with obesity, non-alcoholic fatty liver disease, and cardiovascular disease^1^. Patients with T2D are characterized by multiple metabolic defects including hyperglycemia, insulin resistance, and β-cell dysfunction^2^. During the early stages of T2D, pancreatic β-cells secrete supraphysiological amounts of insulin to compensate for insulin resistance-induced hyperglycemia^3^. However, as the disease progresses, pancreatic β-cells gradually become glucose insensitive and exhibit impaired glucose-stimulated insulin secretion (GSIS), leading to impaired glucose tolerance (IGT)^4^. Although impaired GSIS has been known to be a clear feature of T2D, its pathological causes are still unclear. Kisspeptin is a neuropeptide known to be a prominent regulator of the mammalian reproduction systems^5,6^. In the circulation, kisspeptin undergoes numerous proteolytic processes to form different isoforms including Kp-54, Kp-14, Kp-13, and Kp-10^7,8^. Among these isoforms, Kp-10 was found to be the minimal sequence required for the binding and activation of kisspeptin 1 receptor (Kiss1R, also known as GPR54)^9^. In addition to its regulatory functions in mammalian reproduction systems, kisspeptin, as well as related signaling pathways, may play an important role in the development of T2D. According to Song *et al*., mice and patients with T2D exhibit increased levels of circulatory kisspeptin^10^. More importantly, their data demonstrated that elevated circulating kisspeptin suppresses GSIS from pancreatic β-cells by activating Kiss1R and its downstream signaling pathways^10,11^. Interestingly, previous studies have demonstrated that the concentration determines whether kisspeptin suppresses^12,13^ or activates^14–16^ GSIS. Kisspeptin was usually found to inhibit GSIS at the nanomolar in a GPR54-dependent manner. However, in the micromolar range, kisspeptin usually stimulates GSIS independently of GPR54^17^.

Although many of the signaling pathways involved in the regulation of β-cell insulin secretion remain unclear, recent studies have highlighted the role of autophagy in insulin homeostasis. For instance, disturbed autophagy was identified in the pancreatic β-cells of patients with T2D^18,19^. Moreover, transgenic mice models indicated that hyper-activated autophagy impairs GSIS and IGT^20,21^ while suppressed autophagy increases the proinsulin content of pancreatic β-cells^22^.

Although previous studies have individually identified the important roles of kisspeptin and autophagy in T2D development, none of them have investigated whether kisspeptin impairs insulin secretion by activating autophagy in pancreatic β-cells. Additionally, recent study has demonstrated that kisspeptin directly regulates autophagy in breast cancer cells^23^. Therefore, we hypothesized that long-term exposure of kisspeptin inhibited β-cell insulin secretion by activating autophagy. To test our hypothesis, we overexpressed *Kiss1* in mouse pancreatic β-cells as *in vitro* model and continuously injected kisspeptin to mice as *in vivo* model. In both models, we analyzed autophagy activity and proinsulin and insulin ((pro)insulin) in pancreatic β-cells and measured the changes in insulin secretion.

## Results

### Long-term exposure to kisspeptin inhibits both GSIS and basal insulin secretion in NIT-1 cells

**Figure 1:**
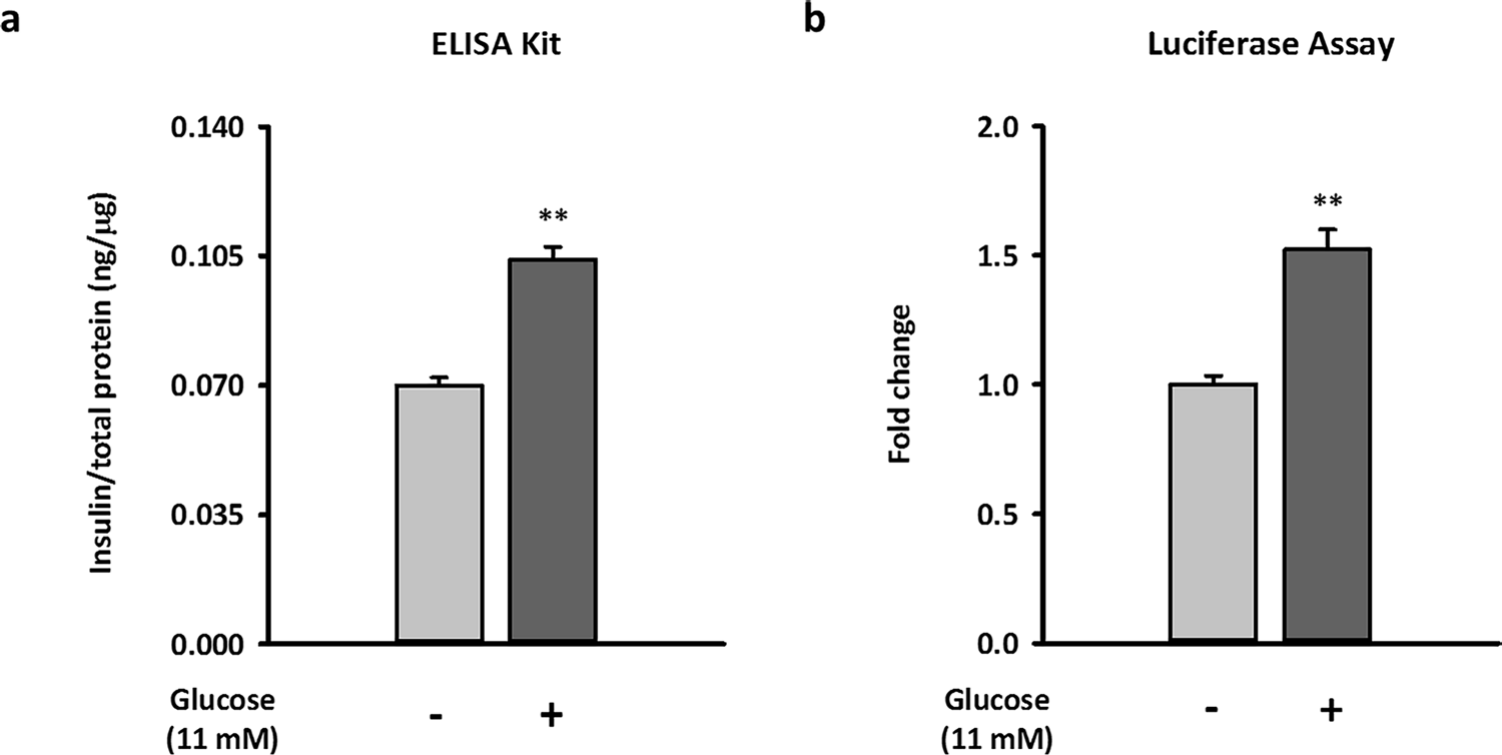
Establishment of the glucose-stimulated insulin secretion model in NIT-1 cells by measuring luminescent activity of luciferase. Equivalent secretion of insulin and luciferase from NIT-1 cells by transfecting pLX304-Proinsulin-NanoLuc were measured. The secretion of insulin (**a**) and luciferase activity (**b**) from the same conditioned media of NIT-1 cells with or without glucose challenge are shown. Both the relative insulin concentration and relative luciferase activity were normalized by total protein in cell lysates. Data represent the means ± standard errors of the mean (n = 3). *Compared with 0 mM glucose treatment; **p < 0.01.

To examine the effects of long-term kisspeptin exposure on β-cell insulin secretion, we first established an *in vitro* GSIS model using the NIT-1 mouse pancreatic β-cell line and a luminescent insulin secretion assay as previously described^24^. Because the pLX304-Proinsulin-NanoLuc plasmid encodes a Gaussia luciferase-inserted mouse insulin C-peptide, the insulin secretion of β-cells expressing this plasmid can be rapidly monitored by measuring luciferase activity in the culture medium. Using this method, luciferase is packaged together with insulin in secretory vesicles and then secreted simultaneously, similar to what C-peptide does naturally. Then, following insulin exocytosis, luciferase is released, allowing for luciferase activity in collected medium monitored and represent the activity of insulin secretion. As shown in Fig. 1, the luminescent assay and conventional enzyme-linked immunosorbent assay (ELISA) methods similarly detected the basal and glucose-stimulated (11 mM) insulin secretion of NIT-1 cells.

**Figure 2:**
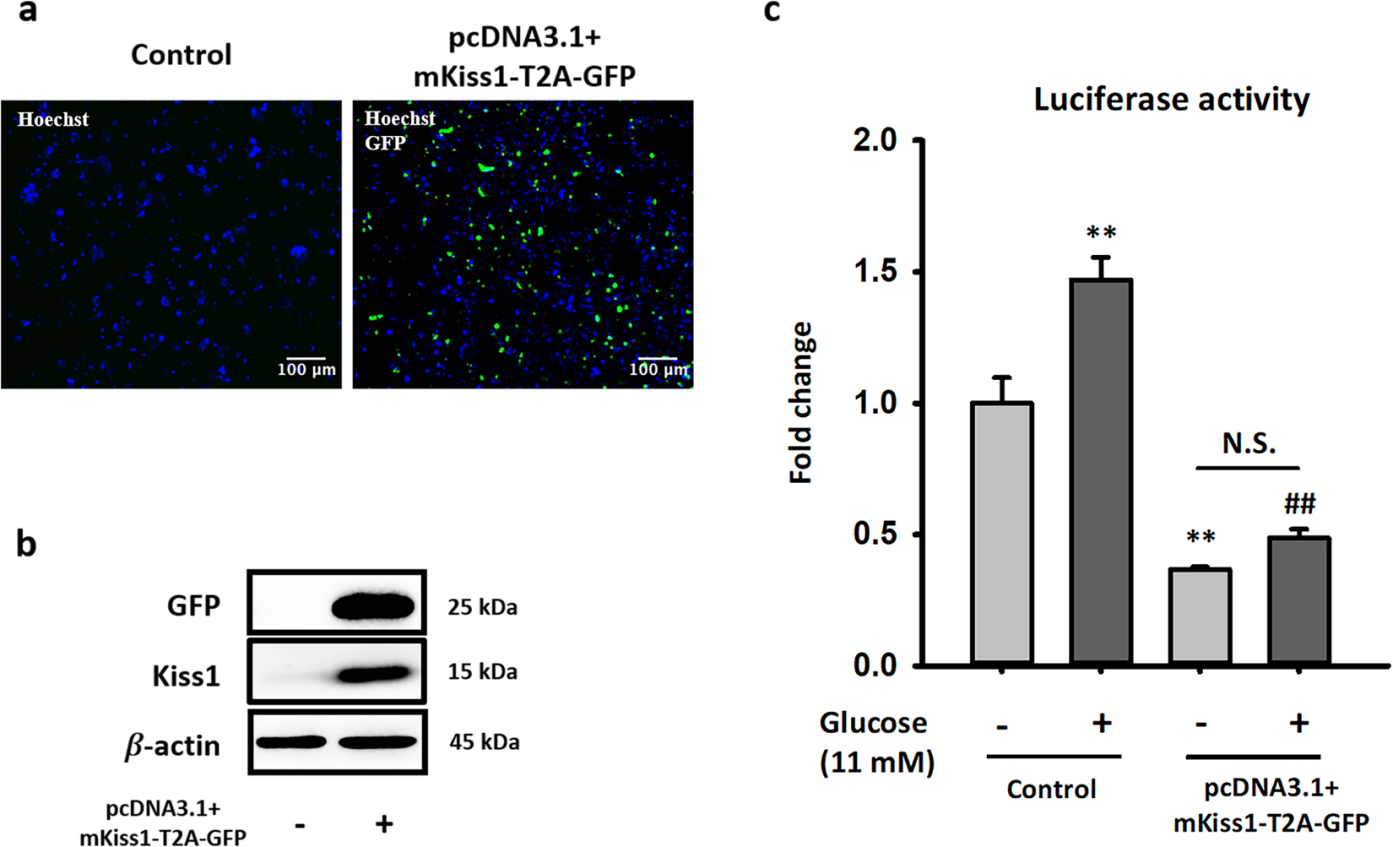
Long-term exposure of kisspeptin inhibits insulin secretion in NIT-1 cells. (**a**) NIT-1 cells were transfected with or without pcDNA3.1 + mKiss1-T2A-GFP plasmid, then fixed and stained with Hoechst 33342. Nucleus and GFP signal are shown in blue and green, respectively. The overlay of signals was processed by ImageJ. (**b**) Representative blots of GFP and mouse kisspeptin from transfected NIT-1 cells are shown. (**c**) Insulin secretion ability of control and transfected NIT-1 cells were determined by the amount of secreted luciferase under basal and glucose-stimulated condition. The relative luciferase activity was normalized by total protein in cell lysates. Data represent the means ± standard errors of the mean (n = 3). *Compared with the basal level in the control group; **p < 0.01; ^#^Compared with glucose-induced level in the control group; ^##^*p* < 0.01. The full-length blots are presented in Supplementary Fig. S1.

Considering the short half-life of kisspeptin in culture medium, we transfected the pcDNA3.1(+)-mKiss1-T2A-plasmid in NIT-1 cells and checked for changes in basal insulin secretion and GSIS. To avoid interference from reporter gene to target gene, T2A was inserted between the sequence of mKiss1 and GFP in our construction. T2A is a 2A peptide from the *Thosea asigna* virus capsid protein. As shown in Fig. 2a, the transfection efficiency of pcDNA3.1(+)-mKiss1-T2A-GFP in NIT-1 cells was approximately 70–80%. Western blot data also showed overexpressed GFP and Kiss1 in transfected NIT-1 cells (Fig. 2b). Importantly, the overexpression of kisspeptin impaired not only GSIS but also basal insulin secretion in NIT-1 cells (Fig. 2c).

### Long-term kisspeptin exposure activates autophagy and decreases (pro)insulin content in NIT-1 cells

**Figure 3:**
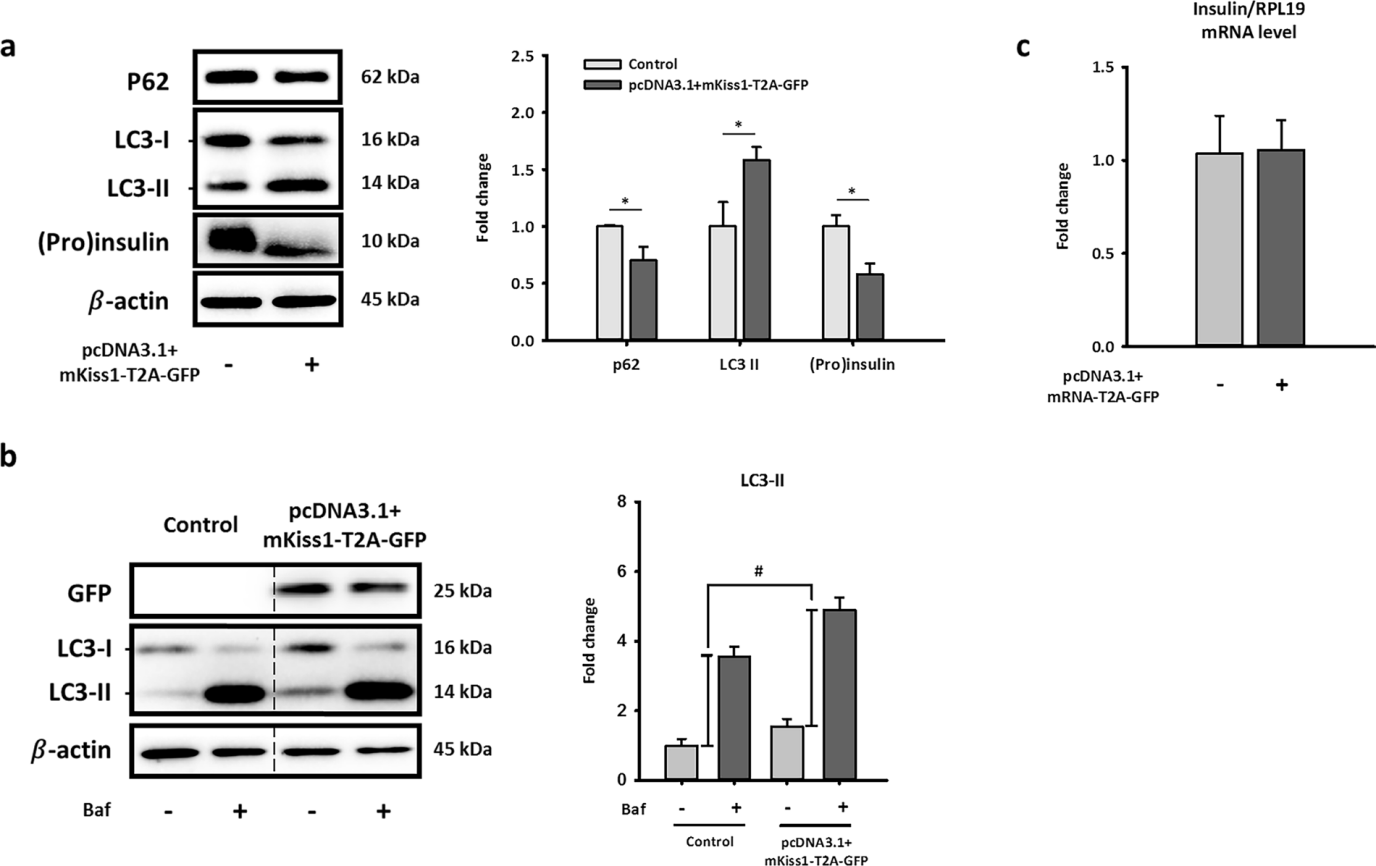
Long-term exposure of kisspeptin decreases (pro)insulin protein level and activates autophagy in NIT-1 cells. Representative blots (**a**) and mRNA levels of insulin (**c**) in NIT-1 cells after transfecting pcDNA3.1 + mKiss1-T2A-GFP for 72 h. Quantifications of blots and mRNA normalized by β-actin or RPL19 are shown as the means ± standard errors of the mean (n = 3). (**b**) Representative blots of autophagy flux marker in cultured NIT-1 cells after overexpressing *Kiss1* with/without treating with bafilomycin A1 (20 nM, 3 h). Quantifications of LC3-II normalized by β-actin are shown as the means ± standard errors of the mean (n = 3), which represents autophagy flux. *Compared with level of the control; ^*^p < 0.05; ^**#**^Compared with autophagy flux of the control; ^**#**^*p* < 0.05. The full-length blots are presented in Supplementary Fig. S2.

To further investigate whether kisspeptin impaired GSIS by activating the autophagic degradation of proinsulin and insulin ((pro)insulin) in pancreatic β-cells, we analyzed the changes of autophagic flux and (pro)insulin content in *Kiss1*-overexpressing NIT-1 cells. Our data showed that long-term exposure to kisspeptin significantly increased the autophagic degradation of (pro)insulin in NIT-1 cells (Fig. 3). The activation of autophagy is indicated by the upregulation of autophagosome marker LC3-II, and the downregulation of p62, which binds to autophagic substrates and delivers them to autophagosome for degradation^25,26^. As shown in Fig. 3a, *Kiss1* overexpression increased LC3-II protein levels while decreasing p62 protein content in NIT-1 cells. The changes in autophagic flux were also confirmed by comparing the amount of LC3 accumulation induced by late-stage autophagy inhibitor bafilomycin A1 in control and *Kiss1*-overexpressing NIT-1 cells (Fig. 3a)^26^. In addition, our western blot data showed that *Kiss1* overexpression significantly decreased intracellular (pro)insulin protein levels in NIT-1 cells (Fig. 3a). Importantly, it is more likely that kisspeptin stimulates (pro)insulin degradation in NIT-1 cells via promoting the pancreatic autophagy rather than inhibiting the biosynthesis of insulin, because the insulin mRNA level in NIT-1 cells was not changed after *Kiss1* overexpression (Fig. 3a). Collectively, the data form Figs 2 and 3 suggested that kisspeptin may suppress insulin secretion from pancreatic β-cells by activating the autophagic degradation of (pro)insulin.

### The activation of autophagy merely contributes to kisspeptin-impaired non-glucose-stimulated insulin secretion from NIT-1 cells

**Figure 4:**
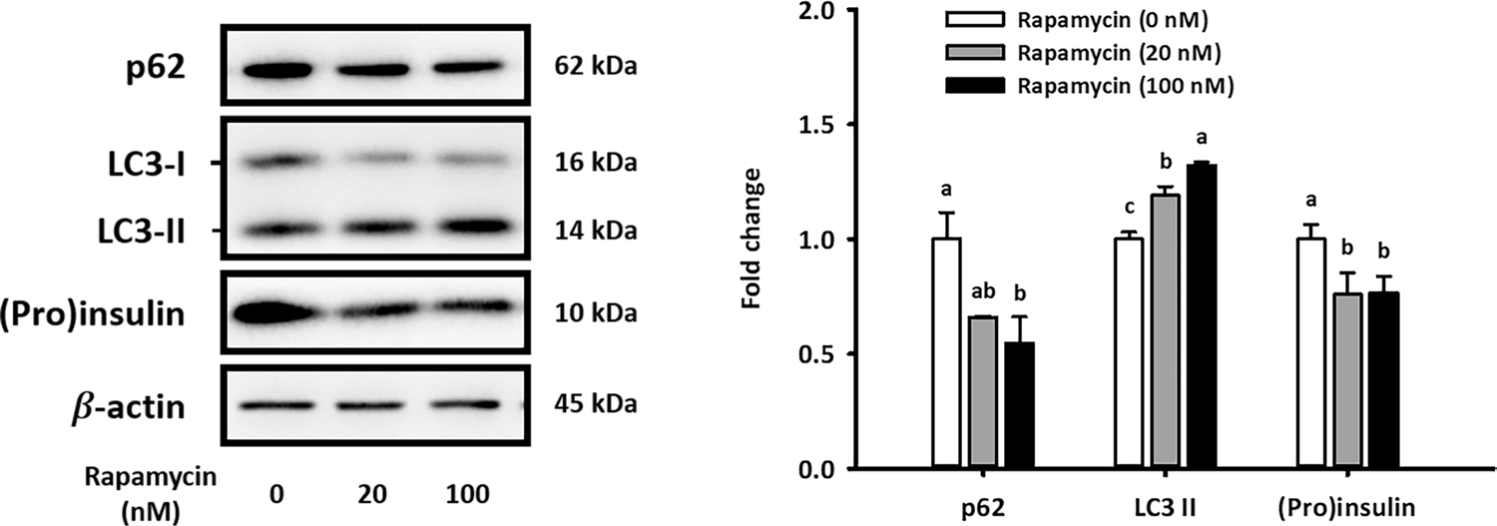
Rapamycin-induced autophagy decreases protein levels of (pro)insulin in NIT-1 cells. Representative blots of autophagy markers and (pro)insulin in cultured NIT-1 after treating with 0 (DMSO), 20, or 100 mM rapamycin for 6 h are shown. Quantifications normalized by β-actin are shown as the means ± standard errors of the mean (n = 3). Different letters represent significant difference determined by one-way ANOVA with post-hoc tests. The full-length blots are presented in Supplementary Fig. S3.

To further investigate whether kisspeptin could suppress β-cell insulin secretion by activating autophagy, we tested whether autophagy activation reduces (pro)insulin content in NIT-1 cells. As shown in Fig. 4, treating with rapamycin, an autophagy activator, significantly increased LC3-II protein expression and decreased p62 content in NIT-1 cells, indicating autophagy activation. Importantly, (pro)insulin content in NIT-1 cells was significantly decreased under activation of autophagy after rapamycin treatment (Fig. 4).

**Figure 5:**
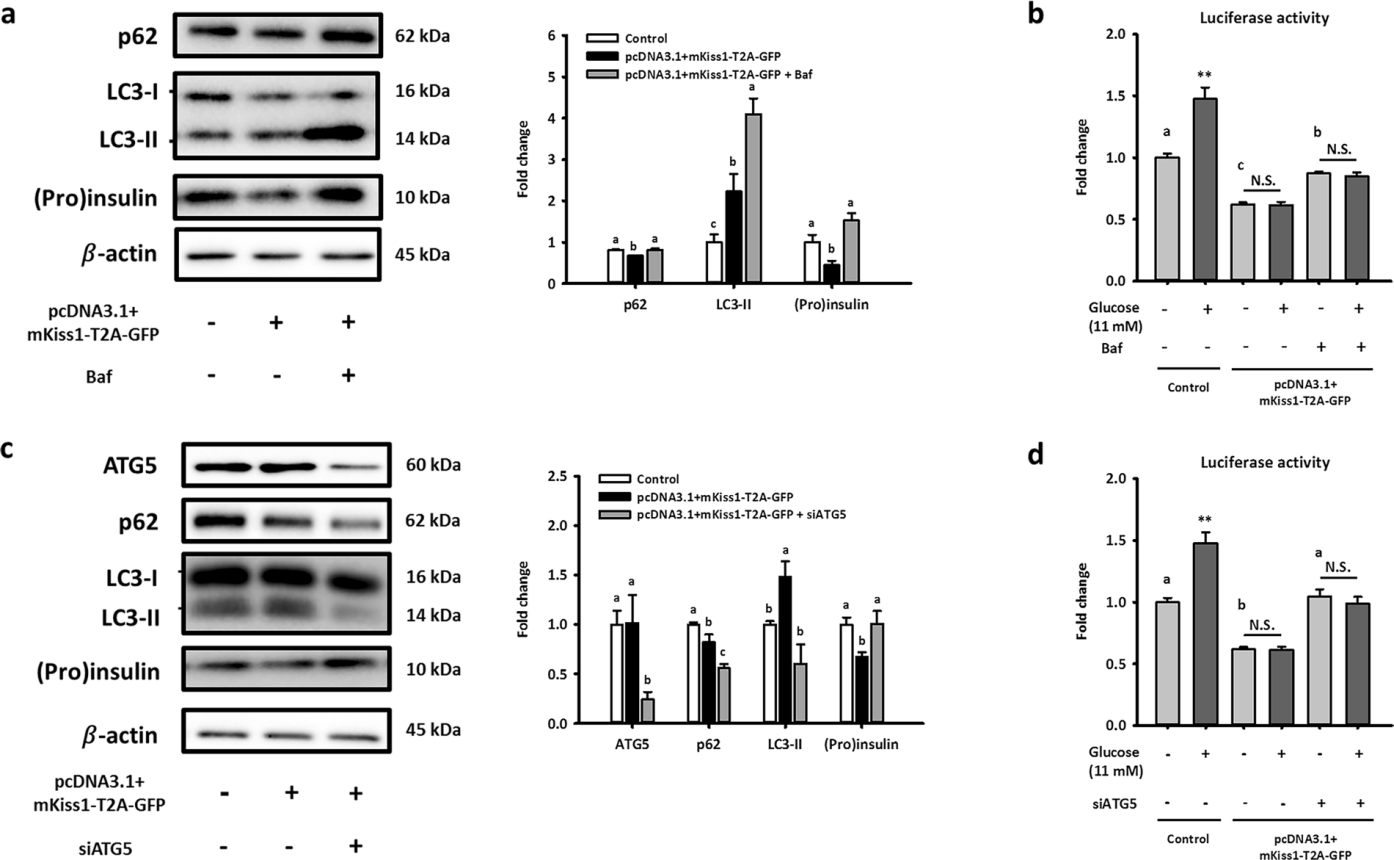
Effects caused by long-term exposure of kisspeptin are partially reversed by inhibition of autophagy in NIT-1 cells. Representative blots of *Kiss1*-overexpressing NIT-1 cells with/without treating bafilomycin A1 (5 nM) for 6 h (**a**) and with/without co-transfecting siATG5 for 48 h (**c**) are shown. Quantifications normalized by β-actin are shown as the means ± standard errors of the mean (SEM) (n = 3). (**b**,**d**) Relative activities of luciferase were normalized by total protein in cell lysates and represent as the means ± standard errors of the mean (n = 3). Different letters represented significant difference determined by one-way ANOVA with post-hoc tests. *Compared with the basal level in the control group; **p < 0.01. The full-length blots are presented in Supplementary Fig. S4.

By using bafilomycin A1 and ATG5 siRNAs to inhibit autophagy, our data further demonstrated that autophagy is required for kisspeptin-reduced (pro)insulin content in NIT-1 cells. As shown in Fig. 5, autophagy inhibition by both bafilomycin A1 and ATG5 siRNA rescued kisspeptin-reduced (pro)insulin content in NIT-1 cells. Collectively, these data confirmed that kisspeptin activates the autophagic degradation of (pro)insulin in NIT-1 cells.

Despite this confirmation, we surprisingly found that autophagy activation only contributed to basal insulin secretion impaired by kisspeptin, not GSIS. As shown in Fig. 5a,c, the reduced basal insulin secretion of *Kiss1*-overexpressing NIT-1 cells was restored by both bafilomycin A1 and ATG5 siRNAs. However, neither bafilomycin A1 nor ATG5 siRNAs rescued the kisspeptin-impaired GSIS from NIT-1 cells (Fig. 5b,d). Taken together, our study clearly demonstrated that autophagy activated by long-term kisspeptin exposure diminishes non-glucose-stimulated insulin secretion from pancreatic β-cells by promoting intracellular degradation of (pro)insulin. However, β-cell GSIS was impaired by other kisspeptin-activated pathways independent of autophagy.

### Long-term injection of Kp-10 inhibits GSIS and decreases pancreatic (pro)insulin in mice

**Figure 6:**
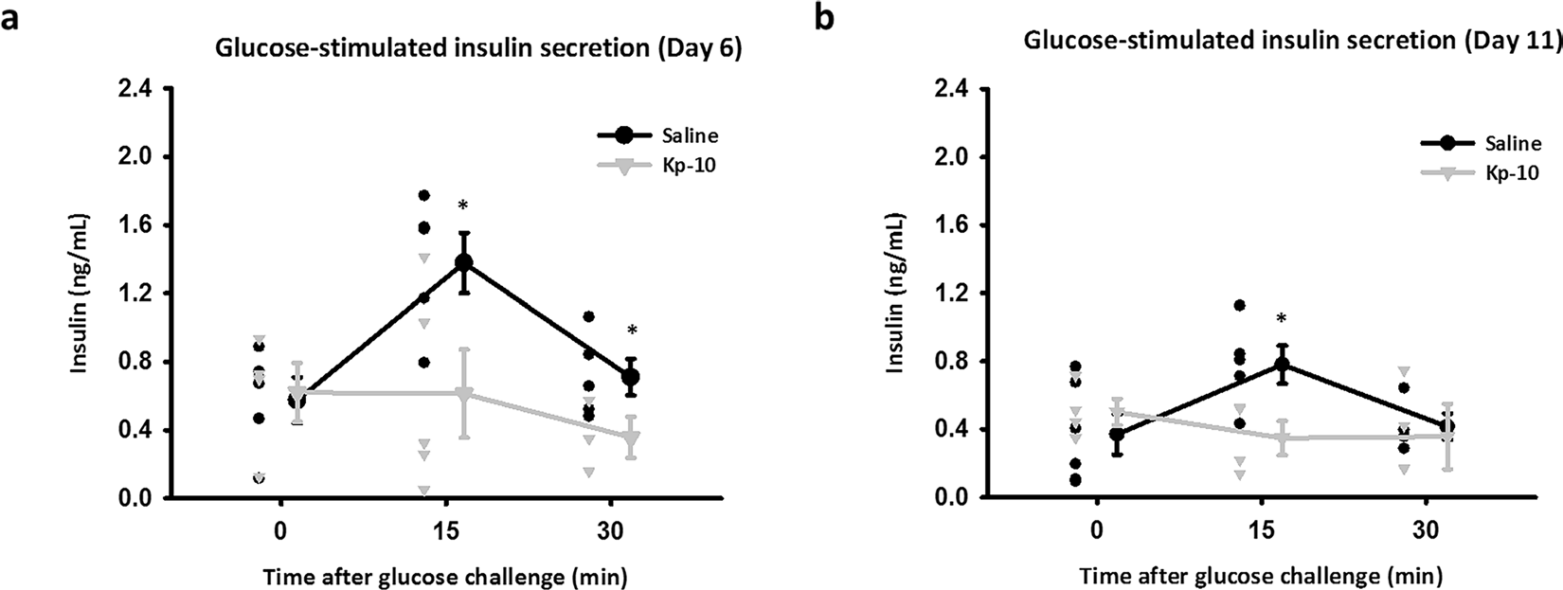
GSIS is inhibited by continuous Kp-10 injection in mice. After implanted osmotic pumps for 6 days (**a**) and 11 days (**b**), serum insulin levels in mice at 0, 15 and 30 min after oral glucose gavage are shown as scatter plots. The circle plots represent the saline group; the triangle plots represent the Kp-10 group (n $\underline{\underline{ > }}$ 3). *Compared with levels of the saline group at indicated time points; *p < 0.05.

**Figure 7:**
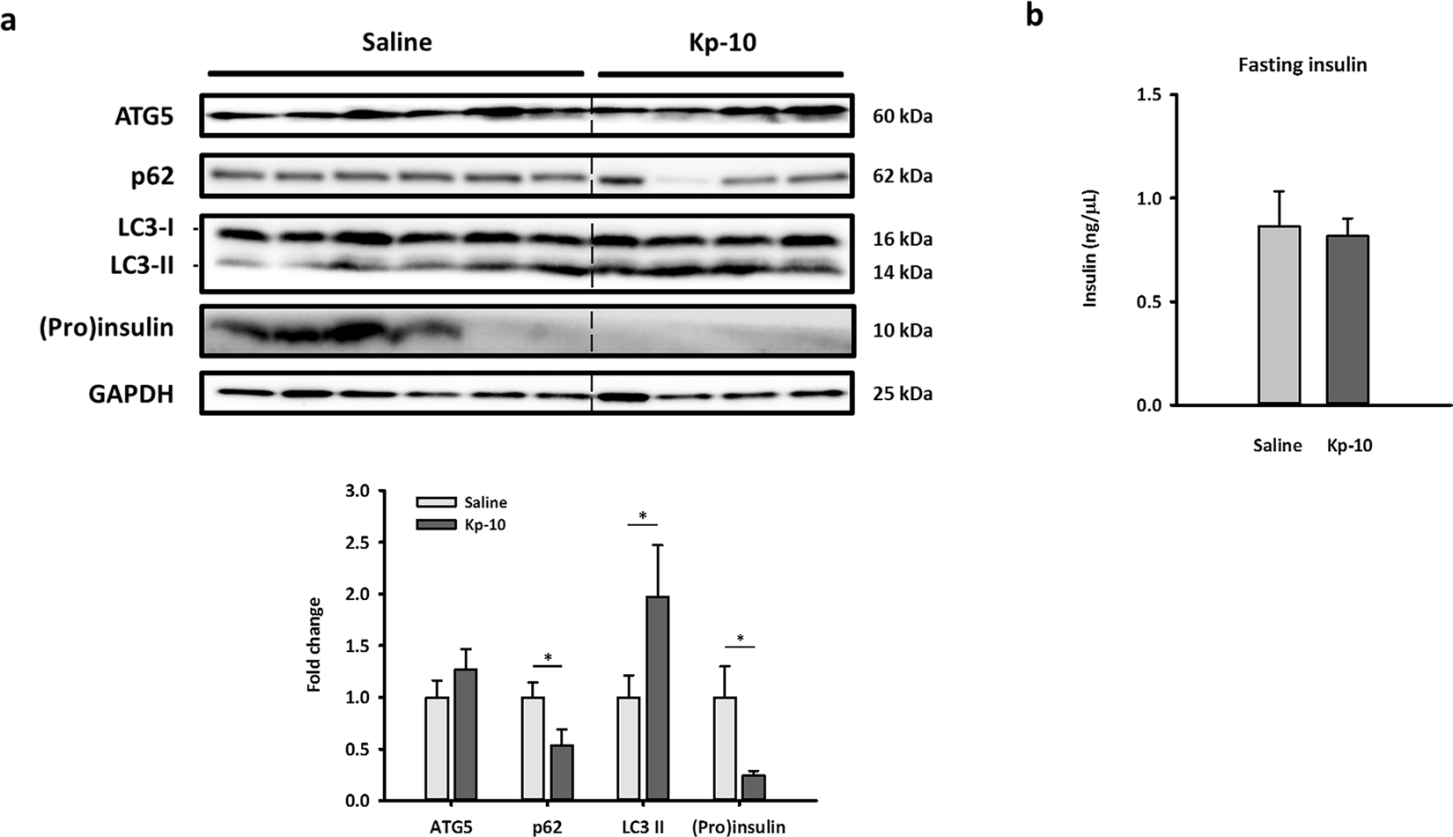
Continuous Kp-10 injection activates pancreatic autophagy and decreases (pro)insulin expression in mice. After implanted osmotic pump for 14 days, blood and pancreas samples were collected under a fasting state. Representative blots of autophagy markers and (pro)insulin in pancreas are shown. Quantifications normalized by GAPDH are shown as the means ± standard errors of the mean (n $\underline{\underline{ > }}$ 4). *Compared with levels of the Saline group; *p < 0.05. The full-length blots are presented in Supplementary Fig. S5.

To confirm the inhibition of insulin secretion by kisspeptin in NIT-1 cells, we further generated an *in vivo* model by exposing mice to Kp-10 (500 ng/h) constantly for 14 days. We also measured GSIS in mice at day 6 and day 11 to assess the long-term effect of Kp-10 on GSIS. After oral gavage of glucose (2 g/kg), GSIS were significantly inhibited by long-term injection of Kp-10 at 15 min (Fig. 6). The results proved that Kp-10 can inhibit GSIS in mice as full-length kisspeptin, similar to those previously described in kisspeptin-overexpressing mice^10^. To further check the involvement of autophagy in insulin secretion, we examined pancreatic autophagy in mice. Similar to *in vitro* data, p62 protein levels decreased and LC3-II protein levels increased in the Kp-10–injected group, suggesting that Kp-10 increases pancreatic autophagy (Fig. 7). Importantly, the expression of (pro)insulin also significantly decreased in Kp-10 treated mice.

## Discussion

Some reports showed that short-term kisspeptin exposure can inhibit GSIS in isolated pancreatic cells^12,13,15^. Although the indirect effects of kisspeptin on the insulin secretions of α and δ cells have been ruled out, it remained unclear how kisspeptin directly affected insulin secretion of β cells^13^. Additionally, it was reported that both patients with T2D and an obese mouse model exhibit increased kisspeptin mRNA in the liver and elevated protein levels in plasma^10^. Taken together, we thought that continuously increased kisspeptin contributes to the development of T2D by inhibiting insulin secretion. In this study, we aimed to evaluate the direct effects of kisspeptin on β-cell insulin secretion and clarify the underlying mechanisms.

To determine whether long-term kisspeptin treatment directly affects insulin secretion in β-cells by regulating autophagy, we employed NIT-1 cell, a mouse pancreatic β-cell line, as our *in vitro* model. NIT-1 cell expresses insulin and GPR54 which is similar to primary mouse islet^27^. Moreover, NIT-1 is much more easily transfected so that NIT-1 is suitable for studying the long-term effects of kisspeptin on insulin secretion through overexpression of *Kiss1*. To measure insulin secretion conveniently and efficiently, we applied the luminescent insulin secretion assay in NIT-1 cells transfected with the pLX304-Proinsulin-NanoLuc plasmid. Although 16.5 mM glucose was previously report to stimulate insulin secretion in NIT-1 cells, it was suggested that NIT-1 cells from different passage number may vary in glucose sensitivity^28^. In our preliminary tests, among all the treatment groups, 11 mM glucose stimulated NIT-1 cells to secrete the highest level of insulin. Therefore, our GSIS model in NIT-1 cells is activated by 11 mM glucose. The application of the luciferase assay in mouse pancreatic β-cell provides a platform for high-throughput screening in insulin secretion, requiring much lesser experimental time and expense than traditional insulin ELISA assay.

An obvious limitation in the investigation of kisspeptin signaling is that the *in vitro* half-life of kisspeptin-10 is 55 second at 37 °C^29^. Due to the difficulty of prolonging the half-life of kisspeptin, we further developed a method to mimic the long-term effects of kisspeptin and aimed to elucidate the possible regulations of kisspeptin in T2D. By transfecting with pcDNA3.1 + -mKiss1-GFP, NIT-1 cells were under long-term stimulation by kisspeptin. Importantly, eukaryotic ribosomes fail to insert a peptide bone between the Gly and Pro residues within the sequence of T2A^30^. Therefore, *Kiss1* and *GFP* were yielded as separate proteins even though they were transcribed together in the same mRNA. By applying the unique plasmid constructed by our lab, we easily and efficiently evaluated transfection efficiency by observing the GFP signal under microscope and measuring indicator proteins by western blot.

As mentioned above, we hypothesized that long-term kisspeptin exposure would lead to impaired GSIS in β-cells. To test our hypothesis, we co-transfected both pcDNA3.1 + -mKiss1-GFP and pLX304-Proinsulin-NanoLuc into NIT-1 cells to mimic long-term kisspeptin exposure and monitor its effects on insulin secretion. Our results showed that the cells overexpressing *Kiss1* would had both reduced basal insulin secretion and GSIS (Fig. 2c). Collectively, long-term kisspeptin exposure generally diminished insulin secretion including GSIS, which may lead to the development of type 2 diabetes. These finding firstly explored the possible mechanisms of non-fuel-stimulated insulin secretion and its involvement in the progression of T2D.

Even though impaired GSIS caused by long-term exposure of kisspeptin to β-cells were clearly demonstrated via transgenic mice^10^, further study of kisspeptin-reduced insulin secretion is needed to illustrate the precise mechanisms at work. The amount of insulin secretion is determined by strict control steps including biosynthesis and degradation^31,32^. Namely, abnormalities in insulin biosynthesis or degradation result in insulin secretion dysfunction due to unbalanced insulin storage. To clarify the major change in β-cells, we measured the mRNA and protein levels of insulin in NIT-1 cells. As the results show in Fig. 3a,c, long-term treatment of kisspeptin significantly decreased protein levels of insulin in NIT-1 cells while the mRNA levels of insulin remained unchanged. These findings suggested that abnormal degradation rather biosynthesis of insulin is the cause for shortage of insulin storage in β-cell, indirectly leading to kisspeptin-impaired insulin secretion.

Recent studies have demonstrated that autophagy plays an important role in insulin degradation in β-cells, but the correlation between autophagy and kisspeptin-impaired insulin secretion in β-cell was not very clear^22^. Interestingly, some reports also showed that diabetes mouse models exhibited increased autophagosomes in β-cells, possibly resulting from induced autophagy^33–36^. Therefore, we wanted to determine whether kisspeptin induces autophagy to degrade more (pro)insulin and thus impair insulin secretion. NIT-1 cell lysates were collected to analyze the marker of autophagy and (pro)insulin content by western blot after overexpressing *Kiss1*. The conversion of LC3-I to LC3-II plays an important role in the formation of autophagosome, and p62, a permanent cargo protein, is responsible for carrying the protein or organelles to the autophagosome^37^. Thus, these two proteins are classically regarded as the targets to monitor autophagy status. When NIT-1 cells overexpressed *Kiss1*, the protein level of p62 decreased while the LC3-II protein increased. Generally, this result is regularly regarded as an indicator of the induction of autophagy because decreased p62 indicates that more targets will be degraded while the increase of LC3-II indicates an increased conversion of LC3-I to LC3-II. Together with the decrease of (pro)insulin content in β-cells, these results seemed consistent with our hypothesis that kisspeptin induces autophagy to degrade (pro)insulin.

Although the pattern of autophagy status shown in Fig. 3a was most consequential of early-stage autophagy induction, it was hard to rule out the possibility of autophagosome accumulation resulting from the inhibition of p62 transcription. Due to the dynamic process of autophagy, other strategies are needed to monitor autophagy^38^ and confirm the clear effect of kisspeptin on pancreatic autophagy. We treated NIT-1 cells with 20 nM bafilomycin A1 for 3 h before sample collection for measuring pancreatic autophagy flux. Because the formation and degradation of the autophagosome is a dynamic process, autophagy flux should be monitored by treating cells with late-stage autophagy blockers. Given that bafilomycin A1 treatment blocks autophagy in the late stage, thus autophagosomes already formed temporarily accumulate in β-cells. Therefore, the accumulation of LC3-II protein levels caused by short-term bafilomycin A1 exposure is regarded as a major indicator of autophagy flux. This higher level of LC3-II protein after bafilomycin A1 treatment means the accumulation of more autophagosomes, suggesting a more thriving autophagy flux. The autophagy flux increased when NIT-1 cells were under long-term exposure to kisspeptin (Fig. 3b). These results indicated that activated autophagy in β-cells is caused by overexpression of kisspeptin. To confirm the decrease of (pro)insulin content in *Kiss1*-overexpressed β-cells indeed resulted from the induction of autophagy, we further treated NIT-1 cells with rapamycin, bafilomycin A1, and siATG5 to regulate pancreatic autophagy. Rapamycin is an inhibitor of mTOR, which is a key suppressor of autophagy in mammalian cells. Our data demonstrated that rapamycin-activated autophagy resulted in decreased (pro)insulin content in NIT-1 cells (Fig. 4), which was similar to the effects of long-term exposure to kisspeptin.

Importantly, our data showed that inhibition of autophagy could reverse the kisspeptin-induced decrease of (pro)insulin content in NIT-1 cells (Fig. 5a). Additionally, basal insulin secretion was also reinstated (Fig. 5b), which confirmed that the observed effects of kisspeptin on basal insulin secretion were due to inducing autophagy. However, unexpectedly, kisspeptin-impaired GSIS remained unrestored (Fig. 5b). These data demonstrated that autophagy plays a novel role in regulating the kisspeptin-impaired basal insulin secretion of pancreatic β-cells, independent of that previously found in kisspeptin-impaired GSIS. Additionally, recent study has shown that autophagy is much more major manner for intracellular (pro)insulin clearance than proteasomal degradation^22^. According to these finding, our data indicated that kisspeptin activates the autophagic degradation of (pro)insulin, contributing to kisspeptin-impaired basal insulin secretion.

Because bafilomycin A1 may inhibit insulin secretion in autophagy-independent ways such as exocytosis, we further assessed the role of autophagy in kisspeptin-impaired insulin secretion using the co-transfection of siRNA targeting the *Atg5* gene. Atg5 is an indispensable protein for LC3-I/LC3-II conversion, which is required for the formation of the autophagosome. Therefore, we transfected siRNA targeting Atg5 to block the kisspeptin-activated autophagy in NIT-1 cells. When Atg5 was significantly silenced, autophagy was inhibited and (pro)insulin content was recovered in NIT-1 cells overexpressing kisspeptin (Fig. 5c). Importantly, similar to the results of co-treatment with bafilomycin A1, insulin secretion levels without glucose stimulation were completely restored under the co-transfection of siAtg5 (Fig. 5d), but no significant difference in insulin secretion was observed between groups overexpressing kisspeptin after glucose stimulation (Fig. 5d). The data shown in Fig. 5 further demonstrated that kisspeptin-activated autophagy in β-cells reduces (pro)insulin content, contributing to suppress only basal insulin secretion, not GSIS.

Prompted by our *in vitro* findings of autophagic regulation in kisspeptin-suppressed insulin secretion, we generated a unique model of long-term kisspeptin injection in mice. Similarly, to overcome the short half-life of kisspeptin, we performed a simple surgery to implant kisspeptin-containing osmotic pumps subcutaneously in mice and compared them with mice implanted with saline-containing pumps. In this model, the pumps release Kp-10 (1 μg/μl) or saline into the mice continuously in a 0.5 μl/h rate for 14 days. Our data demonstrated that long-term injection of Kp-10 significantly inhibits GSIS in mice. Furthermore, kisspeptin also activated pancreatic autophagy and decreased (pro)insulin in mice, similar to the results in NIT-1 cells. However, fasting insulin at different time points (Figs. 6 and 7) in mice were unaffected by our administration of Kp-10. These results may be caused by the duration of fasting time and the difference between *in vivo* and *in vitro* experimental design. Duration of fasting time highly affects fasting and glucose-stimulated insulin level in lean and obese mice^39^. Generally, long fasting leads to low insulin secretion and thus, it is possible that while Kp-10-activated autophagy reduces the (pro)insulin level in β-cells, the remaining insulin content is still able to maintain the same insulin secretion during fasting. In addition, the fasting-activated autophagy may further eliminate the difference of insulin secretion between control and Kp-10-treated mice. Furthermore, insulin secretion assay applied in NIT-1 cell clearly recorded total insulin secretion in 30 minutes, but it could not fully represent fasting insulin level.

Overall, our study demonstrated that induced autophagy resulting from long-term kisspeptin exposure increases the degradation of (pro)insulin in β-cells, which further impairs non-fuel-stimulated insulin secretion. Furthermore, our data suggested that kisspeptin impairs GSIS independently of regulating pancreatic autophagy. We have verified the role of kisspeptin in regulating of insulin homeostasis and basal insulin secretion. However, further experiments are needed to determine how kisspeptin activates pancreatic autophagy and how kisspeptin inhibits GSIS during the development of T2D. Previous studies have shown that kisspeptin signaling through GPR54 inhibits phosphatidylinositol-3-kinase (PI3K)/Akt pathway, negatively regulating mTOR signaling, in tumor cells^40,41^. Further considering inhibition of mTOR signaling activates initiation of autophagy, kisspeptin may activate pancreatic autophagy through the PI3K/Akt/mTOR pathway. Moreover, given that the fasting-induced autophagy was found to preserve β-cell mass in T2D^42^, it is possible that kisspeptin may also protect β-cell against glucolipotoxicity-induced apoptosis by activating autophagy. Because hyperinsulinemia is a common feature of early T2D patients^2^, we consider that the kisspeptin-suppressed insulin secretion may also have protective effects on T2D progression. Collectively, our findings suggest that the kisspeptin-regulated autophagy in pancreatic β-cells plays an important role in T2D. Developing specific kisspeptin agonists targeting β-cell autophagy may provide a potential therapy for T2D patients.

## Methods

### Cell line culture

An NIT-1 cell line was purchased from Taiwan Bioresource Collection and Research Center (BCRC number: 60452). NIT-1 cells were cultured in Dulbecco’s modified Eagle medium (DMEM) (D5030, Sigma-Aldrich) supplemented with 16.5 mM glucose, 2 mM L-glutamine, 15 mM HEPES, 0.02% BSA, 1% penicillin-streptomycin (15140122, Gibco), and 10% heat-inactivated (56 °C for 30 min) and dialyzed fetal bovine serum (10735086001, Roche). Cells were maintained at 37 °C with 5% CO_2_ in a humidified incubator. The NIT-1 cells used in this article were all from passage 38 to passage 47.

### Insulin secretion assay

To simplify the insulin assay process, we applied the luminescent insulin secretion assay as previously described^24^ and compared the results obtained from a traditional insulin ELISA (10-1247-01, Mercodia) assay. Briefly, we conducted the luminescent insulin secretion assay to assess the luciferase activity in culture medium from NIT-1 cells transfected with the purified pLX304-Proinsulin-NanoLuc plasmid, which encodes a Gaussia luciferase-inserted mouse insulin C-peptide fusion protein. To properly mimic stimulation by high glucose, growth DMEM was replaced by DMEM without extra glucose for 24 h before treating with high glucose. In this manner, insulin secretion from NIT-1 cells could be lowered to the basal level and then significantly stimulated by glucose challenge. After 24 h deprivation of glucose, NIT-1 cells were exposed to 11 mM glucose for 30 minutes. After 30-minute glucose challenge, condition medium was collected for further analysis. The pLX304-Proinsulin-NanoLuc plasmid was a gift from David Altshuler (Addgene plasmid # 62057). The collected culture medium was mixed with the coelenterazine luciferase substrate (CZ2.5, GoldBio) and read immediately on a plate reader (Synergy H1 hybrid multi-mode microplate reader, BioTek Instruments Inc.). The activity of luciferase normalized by total protein of cell lysates from each sample was finally identified as insulin secretion.

### Plasmid construction

Our lab constructed the pcDNA3.1(+)-mKiss1-T2A-GFP plasmid containing the sequence of mouse *Kiss1*, the *Thoseaasigna* virus 2 A (T2A), and enhanced green fluorescent (eGFP). The coding sequence in the plasmid can express Kiss1 and eGFP simultaneously and be driven by the human cytomegalovirus (CMV) promoter. The pcDNA3.1(+)-mKiss1-T2A-GFP plasmid was generated first by composing the backbone DNA, the pcDNA3.1(+) mammalian expression vector (Thermo Fisher Scientific), and the insert DNA of mouse *Kiss1*-Hiss-tag. Next, the His-tag fragment of pcDNA3.1(+)-mKiss1-His was replaced by the T2A-GFP fragment amplified from pSpCas9(BB)-2A-GFP (PX458), a gift from Feng Zhang (Addgene plasmid #48138)^43^. The insert DNA sequence was amplified by Phusion High-Fidelity DNA polymerase (Thermo Fisher Scientific) and further cut by FastDigest restriction enzymes (Thermo Fisher Scientific). The DNA ligation was under 1:3 molar ratio of insert: vector DNA using T4 DNA ligase (Takara Bio) at 16 °C for 1 h. Then, the ligated plasmid was transfected to ECOS-101 competent cells (DH5α) (Yeastern Biotech). After that, the bacterial clones were selected by ampicillin-containing agar plates.

### Cell transfection

The purified pcDNA3.1(+)-mKiss1-T2A-GFP plasmids were reverse transfected into NIT-1 cells using Lipofectamine 3000 reagent (L3000015, Thermo Fisher Scientific). To knock down ATG5, predesigned SMARTpool ATG5-specific siRNAs (M-064838-02-0005, Dharmacon) were forwardly transfected into NIT-1 cells via Lipofectamine RNAiMAX (13778150, Thermo Fisher Scientific).

### Autophagic flux assay

To confirm the changes of autophagic flux in NIT-1 cells, we treated the cells with 20 nM bafilomycin A1 (Tocris Bioscience) for 3 h before cell harvest. By comparing the amount of bafilomycin A1-induced LC3-II accumulation in NIT-1 cells, we analyzed the changes of autophagic flux between the group overexpressed *kiss1* and the control group.

### Protein extraction and western blot analysis

Before cell lysis, the cultured cells were washed with phosphate-buffered saline (PBS) once. Then, cells were directly lysed with 1 × Laemmli buffer (2% sodium dodecyl sulfate [SDS], 5 mM DTT, 10% glycerol, 0.002% bromophenol blue, and 63 mM Tris-HCl at pH = 6.8) and boiled at 98° C for 5 min. The proteins with different sizes were then separated by SDS-PAGE using 6–15% gels. The separated proteins were then transferred onto polyvinylidene difluoride (1620177, Bio-Rad) membranes. The membranes were briefly activated in methanol and blocked with 5% milk in TBST (20 mM Tris, 150 mM NaCl, 0.1% Tween-20) for 1 h at room temperature, followed by incubation of target protein-specific antibodies at 4 °C overnight. Then, the membranes were washed three times for 10 min with TBST and hybridized with horseradish peroxidase-conjugated secondary antibodies (in TBST containing 5% milk) at room temperature for another 1 h. After washing three times (5 min) in TBST, the bands on the membranes were visualized by adding the enhanced chemiluminescence substrate (RPN2235, GE Healthcare). All the images were captured under the ChemiDoc gel imaging system (Bio-Rad), and densitometry analysis was performed by Image Lab software (Bio-Rad). The internal controls were applied to normalize the target protein, and the means of quantifications was shown in graphs.

### RNA extraction and qPCR

The total RNA from cultured NIT-1 cells was extracted using the TRIsure reagent (BIO-38032, Bioline) following the manufacturer’s instruction. After RNA extraction, the PrimeScript RT reagent kit (Takara Bio) was applied for the synthesis of first-strand cDNA. To quantify the mRNA expression levels, real-time PCR was performed in a QuantStudio 3 system (Applied Biosystems) using the Fast SYBR Green Master mix (Applied Biosystems) with specific primer pairs listed in Supplementary Table 2. For the calibration of mRNA expression levels, *Rpl19* was used as the internal control.

### Animals

Male C57BL/6 mice were purchased from the Laboratory Animal Center of the College of Medicine at National Taiwan University and were maintained under a 12-h light-dark cycle at 23 ± 2 °C, 40–60% relative humidity with *ad libitum* diet and water. 12-week-old mice were given subcutaneous implants of osmotic pumps (2002, ALZET) to provide continuous delivery of kisspeptin-10 (Kp-10) (500 ng/h) or sterile saline (0.5 μl/h) for 14 days. Kp-10 (H-YNWNSFGLRY-C) were purchased from Kelowna International Scientific Inc. (Taipei, Taiwan). Tests of GSIS in mice were performed on day 6 and day 11. Following 8-h starvation, mice received a single oral dose of glucose (2 g/kg body weight). At 0, 15, and 30 min, blood from the facial vein was collected and analyzed for each mouse. Until day 14, serum and pancreatic samples from both groups of mice were collected for further analysis. All mice were maintained following Guide for the Care and Use of Laboratory Animals^44^, and operations were approved by the National Taiwan University Institutional Animal Care and Use Committee.

### Chemicals and antibodies

Glucose, Hoechst 33342, and bovine serum albumin (BSA) were purchased from Sigma-Aldrich. Bafilomycin A1 and rapamycin was purchased from Tocris. Antibodies listed in Supplementary Table 1 were purchased from Cell Signaling Technology, Abcam, and Santa Cruz Biotechnology.

### Statistical analysis

The statistical analyses were performed, and graphs were plotted using SigmaPlot software 12.0 (Systat Software). All the data were collected from at least three independent experiments and shown as the means ± SEM. Statistically significant differences (*p* < 0.05) were determined by Student’s t-test or one-way ANOVA, followed by Duncan’s multiple range test.

## Supplementary information

Supplementary Information

## Data Availability

All relevant data and resource are available from the authors.
