# Supplementary material for: Kisspeptin-Activated Autophagy Independently Suppresses Non-Glucose-Stimulated Insulin Secretion from Pancreatic β-Cells
Source: Sci Rep. 2019 Nov 25;9:17451. doi: 10.1038/s41598-019-53826-7 (PMC6877614; doi:10.1038/s41598-019-53826-7)
Supplement: Supplementary file 1 — Supplementary Information [file 41598_2019_53826_MOESM1_ESM.pdf]

Kisspeptin-Activated Autophagy Independently Suppresses Non-Glucose-Stimulated Insulin Secretion from Pancreatic β-Cells

Chien Huang[1, #], Hao-Yi Wang[1, #], Mu-En Wang[1, 2], Meng-Chieh Hsu[1], Yi-Hsieng Samuel Wu[1], Yi-Fan Jiang[3], Leang-Shin Wu[1], De-Shien Jong[1], and Chih-Hsien Chiu[1, *]

[1]Laboratory of Animal Physiology, Department of Animal Science and Technology, National Taiwan University, Taipei 10617, Taiwan

[2]Department of Pathology, Duke University School of Medicine, Duke Cancer Institute, Duke University, Durham, NC 27514, USA.

[3]Graduate Institute of Molecular and Comparative Pathobiology, School of Medicine, National Taiwan University, Taipei 10617, Taiwan

[#]Equal contribution

***Corresponding Author: Chih-Hsien Chiu**

Laboratory of Animal Physiology, Department of Animal Science and Technology

National Taiwan University

No. 50, Lane 155, Section 3, Keelung Road, Taipei City 106, Taiwan

Tel.: +886-2-3366-4171; Fax: +886-2-3366-4070; Email: chiuchihhsien@ntu.edu.tw

Supplementary Tables

Table 1. Antibodies used in Western Blotting

Antibody Name	Company	Product Number	Dilution
Anti-GFP	Santa Cruz Biotechnology	sc-9996	1:1000
Anti-Kiss1	Cloud-Clone Corp	PAC559Mu01	1:400
Anti-β-actin	Santa Cruz Biotechnology	sc-47778	1:2500
Anti-SQSTM1/p62	abcam	ab109012	1:1000
Anti-LC3	Cell Signaling Technology	#2775	1:1000
Anti-insulin	Cell Signaling Technology	#8138	1:1000
Anti-ATG5	Cell Signaling Technology	#12994	1:1000
Anti-GAPDH	Cell Signaling Technology	#2118	1:2500
Anti-PARP	Cell Signaling Technology	#9532	1:1000
Anti-Cleaved Caspase 3	Cell Signaling Technology	#9661	1:250

Table 2. Primers used in qPCR analyses

Gene Name	Forward (5' to 3')	Reverse (5' to 3')
Insulin	GCAGAGAGGAGGTACTTTGGA	GGTAGGAAGTGCACCAACAG
RPL19	GCTCTTTCCTTTCGCTGCTGC	CAGTCACAGGCTTGCGGATGAT

Supplementary Figure Legends

Supplementary Figure S1. The full-length blot of GFP, Kiss1, β-actin presented in Figure 2 of the main text.

Supplementary Figure S2. The full-length blot of GFP, p62, LC3, Proinsulin, β-actin presented in Figure 3 of the main text.

Supplementary Figure S3. The full-length blot of p62, LC3, Proinsulin, β-actin presented in Figure 4 of the main text.

Supplementary Figure S4. The full-length blot of ATG5, p62, LC3, Proinsulin, β-actin presented in Figure 5 of the main text.

Supplementary Figure S5. The full-length blot of ATG5, p62, LC3, Proinsulin, β-actin presented in Figure 7 of the main text.

Supplementary Figure S6. The dosage response of the glucose-stimulated insulin secretion in NIT-1 cells by measuring luminescent activity of luciferase. After 30-minute collection, the insulin secretion from NIT-1 under 0, 5.5, 11 and 16.5 mM glucose challenge was measured by luciferase activities. Quantifications normalized by total protein in NIT-1 cells are shown as the means ± standard errors of the mean (n = 3). Different letters represent significant difference determined by one-way ANOVA with post-hoc tests.

Supplementary Figure S7. The bafilomycin treatment does not trigger apoptosis in NIT-1 cells. Representative blots of apoptosis markers in NIT-1 cells after treating with 0, 1, 5, or 20 nM bafilomycin for 6 h; quantifications normalized by β-actin are shown as the means ± standard errors of the mean (n = 3). Indicated markers have no significant differences determined by one-way ANOVA.

Supplementary Figure S8. Short-term exposure of kisspeptin decreases (pro)insulin level and activated autophagy in NIT-1 cells. After transfecting pcDNA3.1+mKiss1-T2A-GFP plasmid for 66 h, the growth media for NIT-1 cells were changed to blank media for another 6-hour culture. After 6-hour culture, the conditioned media from control and *Kiss1*-overexpressing group were collected and then treated to the non-treated NIT-1 cells for 2 minute. Then, the conditioned media-treated cell lysates from two group were collected for further analysis. The treated conditioned media to control group were collected from NIT-1 cells transfected with reagent only. Representative blots of autophagy markers and (pro)insulin in short-term treated NIT-1 cells and quantifications normalized by GAPDH are shown as the means ± standard errors of the mean (SEM). *compared with the control; *$p < 0.05$, **$p < 0.01$.

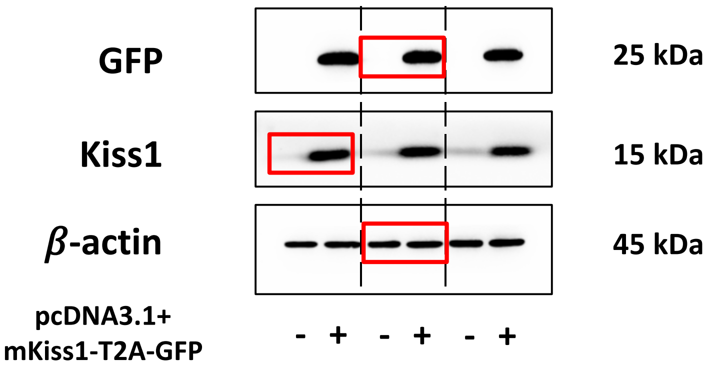

Supplementary Figure S1

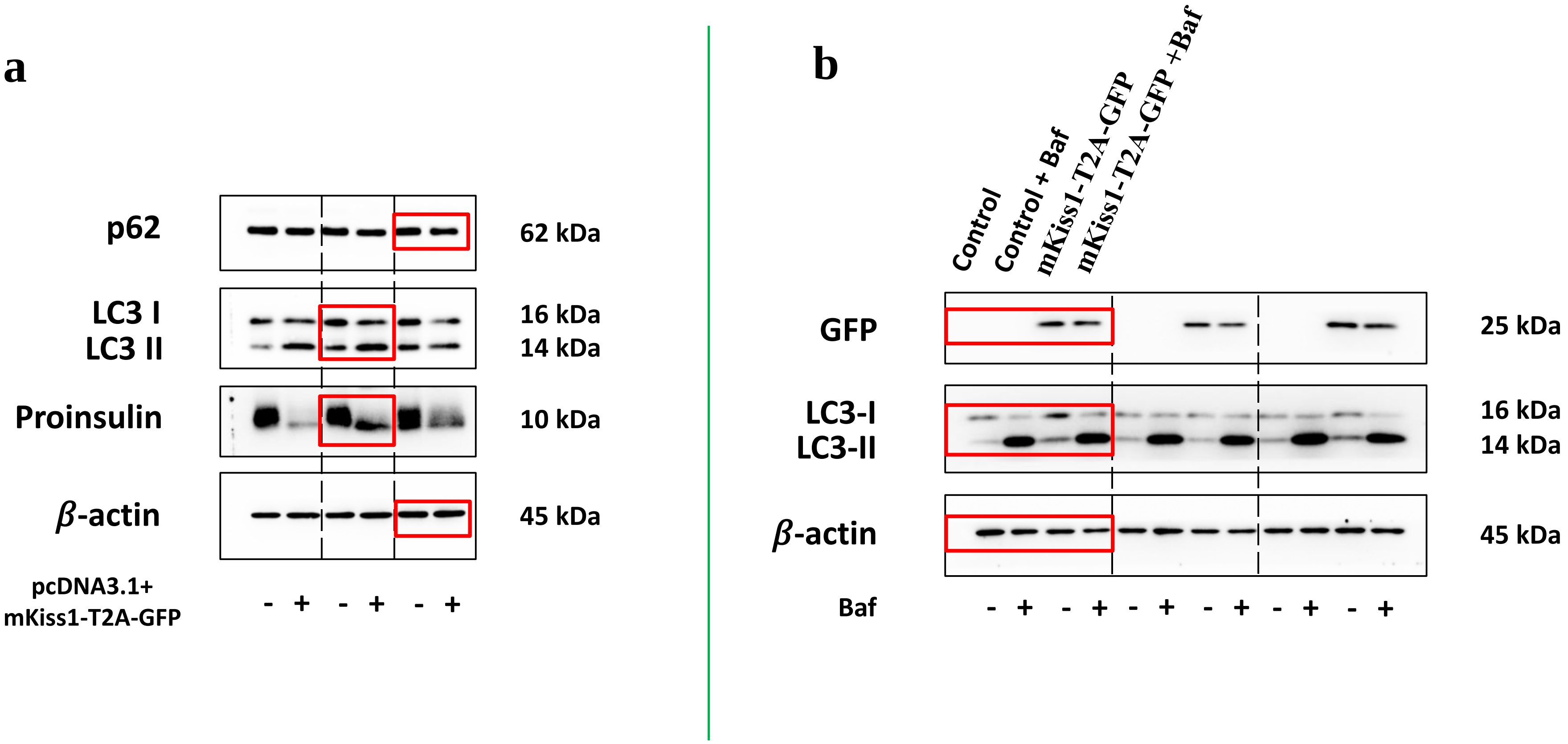

Supplementary Figure S2

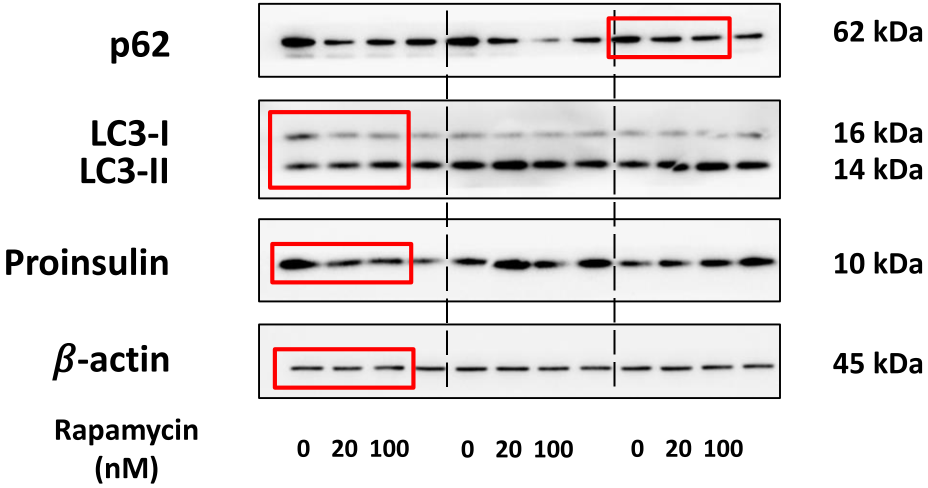

Supplementary Figure S3

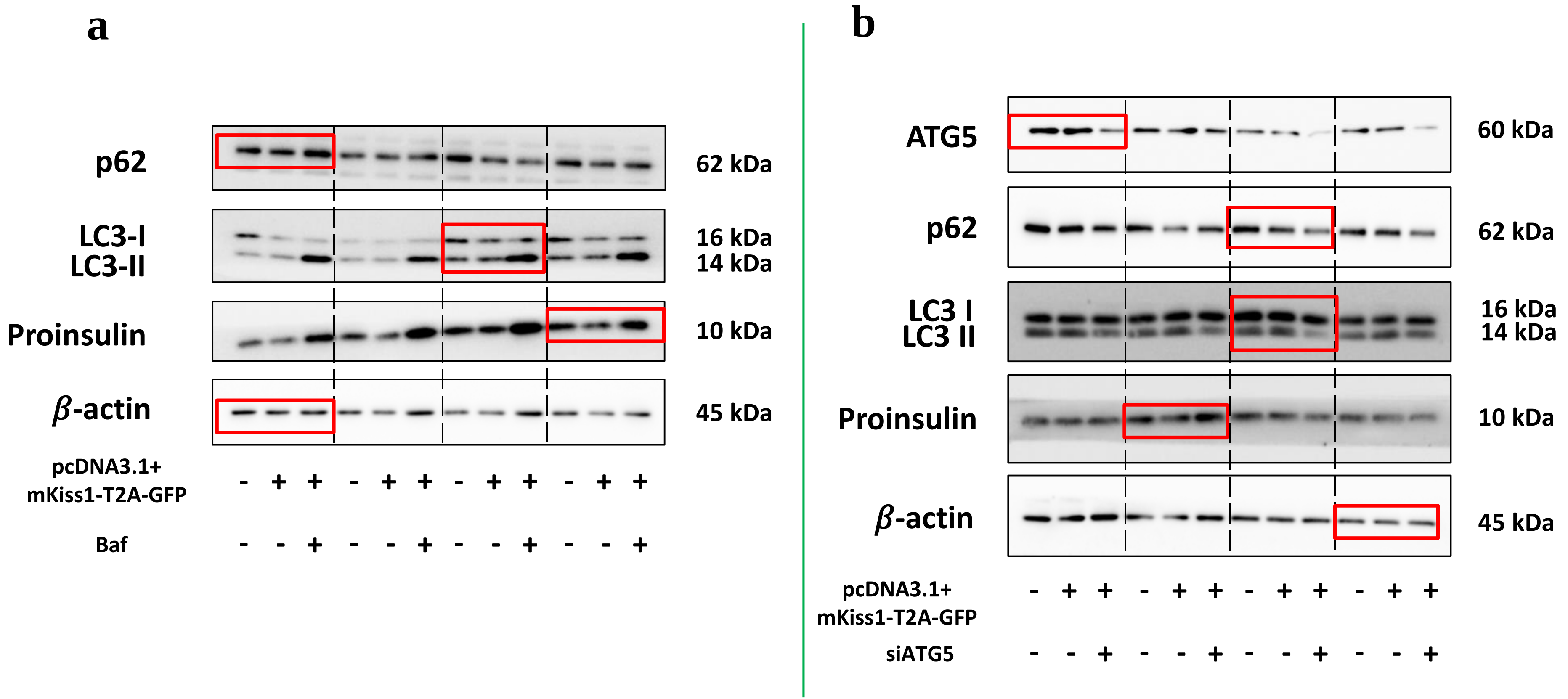

Supplementary Figure S4

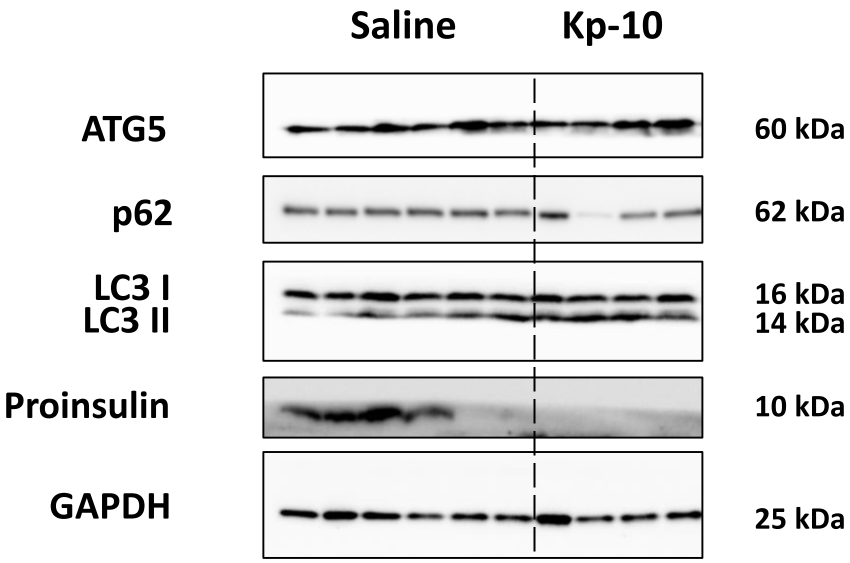

Supplementary Figure S5

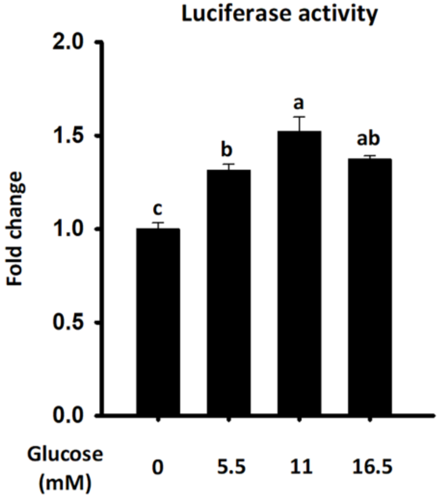

Supplementary Figure S6

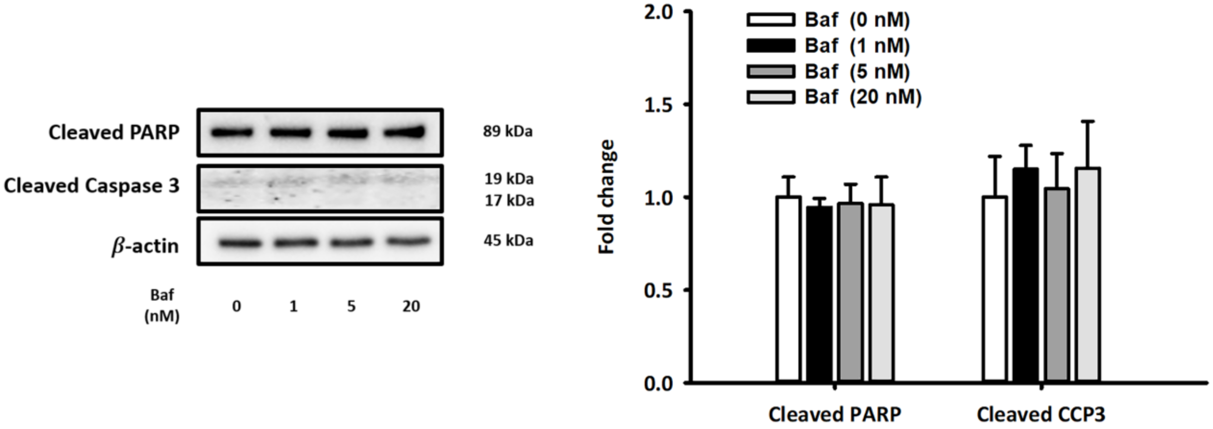

Supplementary Figure S7

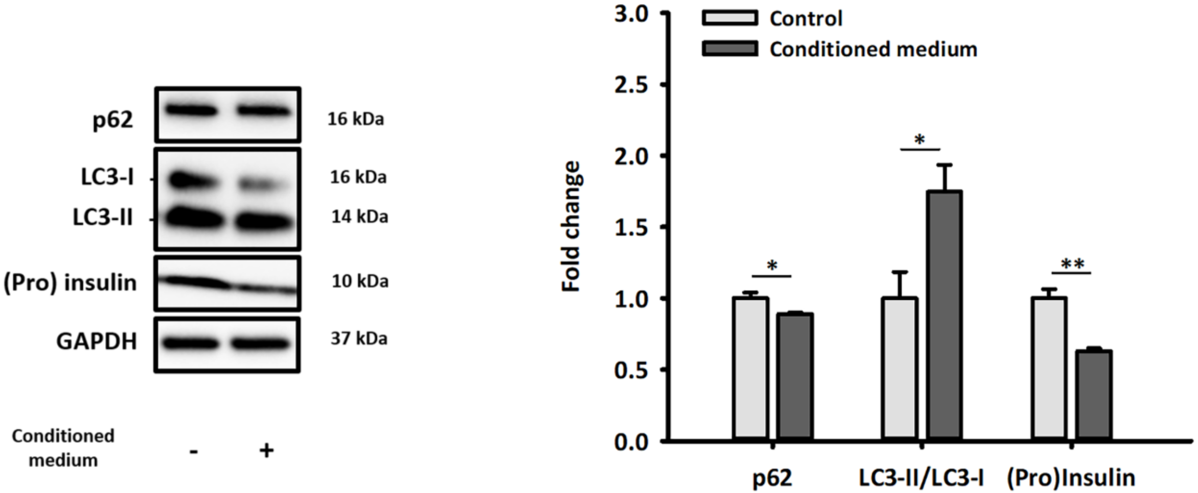

Supplementary Figure S8
